# Psychosocial Factors Associated with Sleep Quality and Duration Among Older Adults with Chronic Pain

**DOI:** 10.1089/pop.2019.0165

**Published:** 2021-02-02

**Authors:** Catherine Zaidel, Shirley Musich, Jaycee Karl, Sandra Kraemer, Charlotte S. Yeh

**Affiliations:** ^1^ Research for Aging Populations, Optum, Ann Arbor, Michigan, USA.; ^2^ UnitedHealthcare Medicare and Retirement, Minneapolis, Minnesota, USA.; ^3^ AARP Services, Inc., Washington, District of Columbia, USA.

**Keywords:** resilience, social networks, Pittsburgh Sleep Quality Index

## Abstract

Sleep complaints are common among older adults with chronic pain. Because of the risk of significant side effects, sleep medications are not recommended as first-line treatments. Little is known about the association between positive psychosocial factors and sleep, but further awareness could support non-drug strategies to minimize poor sleep. The purpose of this study was to (1) determine the prevalence of self-reported poor sleep quality and short/long sleep duration in a population of older adults with chronic pain, and (2) examine the associations of negative risk factors, sleep-inducing medications, and positive psychosocial characteristics on sleep outcomes in this population. This study analyzed survey responses from 4201 adults ages ≥65 years with diagnosed back pain, osteoarthritis, and/or rheumatoid arthritis, and at least 1 year of continuous medical and drug plan enrollment. The most commonly reported sleep outcome was short sleep duration (39%), followed by poor sleep quality (22%), and long sleep duration (9%). Based on pharmaceutical claims, prescriptions for opioids (59%) or benzodiazepines (22%) were common. Perceived stress, depression, and pain or sleep prescription medications were independently associated with poor sleep quality and short or long sleep durations. The positive psychosocial factors of higher resilience and more diverse social networks were independently associated with good sleep quality and optimal sleep duration. These results underscore the importance of social and coping factors to sleep, which may provide new opportunities to improve sleep and well-being in older adults with chronic pain.

## Introduction

Sleep is a complex behavior associated with a range of factors, including physical and mental health, medication use, and psychosocial characteristics.^1,2^ Sleep complaints are common among older adults; approximately half of those older than age 65 in the United States report trouble falling asleep, staying asleep, or awakening too early or without feeling rested.^2–4^ Although sleep problems are common among older adults, numerous population-based studies have found that they are not independently associated with chronological age.^1^ Rather, such sleep problems often are associated with other age-related changes, namely chronic conditions, pain, anxiety, depression, and negative psychosocial factors such as social isolation and loneliness.^1,5^ Thus, the prevalence of sleep complaints is high among older adults because the prevalence of factors adversely affecting sleep increases with age.^2,4^

In the absence of comorbid conditions, normative age-related sleep changes are minimal,^4^ as reflected in the National Sleep Foundation's (NSF) recent guidance on healthy sleep quality and quantity across the life span.^6,7^ “Sleep quality” has been defined and measured in myriad ways; however, the consensus among experts is that it reflects sleep continuity.^6^ Subjective assessments of sleep quality indicate how satisfied an individual is with his/her sleep, whereas objective measurements of sleep quality include sleep latency, number of awakenings, time awake after sleep onset, and sleep efficiency.^6^ These objective measurements may worsen among older adults, but normative changes are modest.^6^ Optimal sleep duration among older adults is considered to be approximately 7–8 hours per night.^7^ Both shorter and longer sleep durations relative to these recommendations have been associated with increased incidence of cardiovascular disease, diabetes mellitus, stroke, and all-cause mortality.^8–12^ Because sleep plays an important role in the regulation of inflammatory responses, adverse health outcomes from poor quality or short sleep are believed to be associated with increased systemic inflammation.^13^ The nature of the association between long sleep duration and increased mortality risk remains poorly understood, and it is unknown whether these associations are causal or modifiable.^8,11,12^

Until recently, clinicians and researchers often overlooked sleep complaints among older adults as symptoms of coexisting health conditions.^14^ Restorative sleep is now recognized as essential for physical, cognitive, and psychological well-being across the life span.^4,9,13,15–17^ Sleep itself is an important behavioral risk factor for worsening pain, depression, anxiety, and chronic conditions, perpetuating both sleep problems and poor health outcomes. Thus, unresolved sleep problems have far-reaching consequences on physical and mental health among older adults.^1,11^ For older adults with chronic pain conditions, sleep is particularly important.^18^ Whereas pain is a well-recognized risk factor for the development of new-onset sleep problems,^1,16^ several studies have found that sleep is a stronger predictor of future pain sensitivity than pain is of future sleep problems.^15,17^ Thus, identifying and treating sleep disturbance may provide important benefits to pain management in older adults.^15,18^

Psychiatric conditions further complicate the relationship between sleep disturbance and pain in older adults. For example, sleep disturbance is a well-known predictor of new-onset or worsening depression and anxiety.^3,15^ A recent meta-analysis found that the risk of new-onset or worsening depression was nearly 4 times greater among older adults with persistent sleep disturbance.^3^ Likewise, the authors reported that depression increased the risk of new or worsening sleep disturbances by about by 70%.^3^ Among older adults with chronic pain conditions, a recent, nationally representative longitudinal study found that anxiety mediated up to 17% of the total effect of insomnia on incident pain. However, depression was not observed to significantly modify the relationship between sleep and pain in older adults.^15^

Sleep complaints among older adults are frequently treated with sleep-inducing medications, despite contraindications for use among older adults because they hinder daytime vigilance, increase fall risk, and compromise cognitive function.^19^ Despite these well-recognized risks, older adults are twice as likely as younger adults to be prescribed sleep-inducing medications such as benzodiazepines, non-benzodiazepine receptor agonists, and antipsychotics.^9,19,20^ Not only are these medications associated with considerable adverse effects, but benefits are modest at best.^9^

Recent research into positive psychosocial resources suggests possible non-pharmacologic intervention strategies that build on these positive resources. Previous studies have reported positive associations between higher resilience, more diverse social networks and improvements in chronic pain, depression, and overall well-being in older adults. Few studies have examined the relationship between positive psychosocial resources and sleep among older adults. Cross-sectional analyses in a study published by Chen et al found positive associations between social participation and objectively-measured sleep among adults older than age 57.^21^ No study to date has assessed the association between resilience and sleep in older adults.^21–24^

Meanwhile, several studies have examined correlates of sleep complaints among older adults, but these efforts have not captured the full picture of physical and mental illness, medication use, and psychosocial factors. Thus, the primary objectives of this cross-sectional study were to (1) investigate the prevalence of poor sleep quality and short/long sleep duration among older adults with chronic musculoskeletal pain, and (2) estimate the effects of negative risk factors, medications, and positive psychosocial factors on these sleep outcomes. This study was covered under New England Independent Review Board (NEIRB #120160532).

## Methods

### Study population

All study participants were covered under AARP^®^ Medicare Supplement Insurance Plans and AARP MedicareRx Plans, insured by UnitedHealthcare Insurance Company (for New York certificate holders, UnitedHealthcare Insurance Company of New York). These plans are offered in all 50 states, Washington DC, and various US territories. Eligible participants included insureds ages ≥65 years, with at least 1 year of continuous enrollment in medical and drug plans having a diagnosis of low back pain, osteoarthritis, and/or rheumatoid arthritis, based on *International Classification of Diseases, Tenth Revision, Clinical Modification* codes from health care claims. Those with cancer, trauma, or drug abuse were excluded. The stratified mailing list of 15,000 was drawn from a national sample of 327,685 eligible insureds to include 7500 with back pain, 5000 with osteoarthritis, and 2500 with rheumatoid arthritis. Survey respondents (4423, 29% response rate) who did not match eligibility files (N = 50) and those who did not answer questions about sleep quality and duration (N = 172) were excluded. Thus, the final study population included 4201 (95% of respondents).

### Survey

UnitedHealthcare designed a 54-question survey to evaluate physical and psychosocial factors associated with well-being among AARP Medicare Supplement insureds and AARP MedicareRx plan holders with chronic pain. This survey assessed sleep quality and duration, body mass index (BMI), resilience, social networks, perceived stress, and depression. The survey was mailed to the stratified sample in May 2018. A second survey was mailed in June 2018 to those who had not responded.

### Sleep outcomes

Sleep quality and duration were measured with questions from the Pittsburgh Sleep Quality Index.^25^ Sleep quality was assessed with the question: During the past month, how would you rate your sleep quality overall? Out of 4 possible responses, *fairly bad* and *very bad* were used to define poor sleep quality. Sleep duration was assessed with the question: During the past month, on an average night, how many hours of actual sleep did you get at night? Based on NSF recommendations for older adults, short, optimal, and long sleep durations were defined as ≤6, 7 to 8, and ≥9 hours per night, respectively.

### Perceived stress and depression

Perceived stress was measured using 4 validated questions from the Perceived Stress Scale, in which respondents reported how often during the last month they felt their life was unpredictable, uncontrollable, or overloaded.^26^ Responses for each question ranged from zero to 4 and were summed to give an overall perceived stress score ranging from zero to 16. Scores were calculated for respondents who answered at least 2 of the 4 questions. Perceived stress was stratified into 3 categories: low (scores of 0 to 5), moderate (scores of 6 to 10), and high (scores ≥11).

Depression was identified using the validated 2-item Patient Health Questionnaire-2 (PHQ-2).^27^ Respondents answered 2 questions about how often they experienced depressed mood and loss of interest during the previous 2 weeks. Responses for each question ranged from zero to 3 and were summed to give an overall score ranging from zero to 6. Depression was defined as a score of ≥3. Scores were calculated if at least 1 of the PHQ-2 questions was answered.

### Positive psychosocial characteristics

Resilience was measured with the 6-item Brief Resilience Scale,^28^ and scores were calculated for respondents who answered at least 3 of the 6 questions. Responses for each question ranged from 1 to 5, and the resilience score was calculated as the average of the questions answered. Average scores of 4 and higher (scores ≥4; corresponding to responses agree and strongly agree) were considered high resilience and scores less than 4 (score <4) were considered low resilience.

The Social Network Index measures how often respondents interact with family and friends over the telephone or in person each week, as well as the frequency of participation in religious services or other group activities each month.^29^ Responses to each of 4 questions ranged from 0 to 3. One additional point was given to respondents who were married or living together with someone in a partnership at the time of response. Thus, total social network scores ranged from zero to 13 and were stratified into 3 categories: limited (scores of 0 to 4), medium (scores of 5 to 7), and diverse (scores ≥8).

### Prescription drug use

Pharmaceutical claims data for the 12 months before May 2018 were reviewed to identify National Drug Codes for medications in 6 specific therapeutic classes, including antipsychotics, benzodiazepines, muscle relaxants, nonsteroidal anti-inflammatory drugs, opioids, and sleep medications.

### Covariates

Demographic and health status covariates were obtained from a combination of survey and administrative claims and used to control for the effects these characteristics may have on sleep quality and duration outcomes.

Age, sex, and health plan type were obtained from administrative data. Geographic region (Northeast, South, Midwest, and West), metropolitan location (urban, other), percent nonwhite population (low, medium, high), median household income (low, medium, high), and health care supply (primary care physicians per 100,000) were derived by linking zip codes to census data. AARP Medicare Supplement plan types were grouped into 3 categories by level of cost sharing with the lowest co-payments and deductibles considered as high-level coverage, followed by medium-level coverage, and all other plans.

Health status is typically represented by members' Hierarchical Condition Category (HCC) scores. These scores are calculated using a model developed by the Centers for Medicare & Medicaid Services to estimate future medical expenditures for risk-adjusted payments across medical plans.^30^ The HCC model calculates scores on the basis of an individual's age, sex, and chronic medical conditions and can be used as a proxy measure for health status, with higher scores indicating poorer health status. HCC scores were categorized into 4 groups: <0.5, 0.5 to <1.2, 1.2 to <2.8, and ≥2.8.

Self-reported height and weight were used to calculate BMI. Individuals were grouped into 3 standard categories: healthy weight (BMI <25), overweight (BMI = 25 to <30), and obese (BMI ≥30).

### Statistical models

Propensity weighting procedures were used to address potential survey nonresponse bias.^31,32^ Population weights also were applied to adjust for oversampling so that results were generalizable to the population of AARP Medicare Supplement insureds with back pain, osteoarthritis, and/or rheumatoid arthritis chronic pain.

Medical and psychosocial characteristics associated with sleep were evaluated with multivariate logistic regression models using the propensity and population-weighted data. Perceived stress and depression were highly correlated (Spearman rho = 0.42) and were analyzed in separate models. The basic regression models included the demographic (age, sex, minority status, location, region), socioeconomic (income, plan type), and health status (HCC score categories, obesity, low back pain, osteoarthritis, and rheumatoid arthritis) variables. Independent variables, including perceived stress, depression, prescription medications, resilience, and social networks were added 1 at a time to the basic model. All variables were included in the full models, with depression and stress evaluated separately. Covariate variables with high correlations (>0.5) were dropped from the models. All analyses were completed using SAS Enterprise Guide Version 7.1 (SAS Institute Inc., Cary, NC, USA).

## Results

Among AARP Medicare Supplement insureds who received a survey, 4373 responded (29% response rate), and 4201 (96%) met eligibility criteria and were included in this study. In the unadjusted sample, the prevalence of low back pain, osteoarthritis, and rheumatoid arthritis was 56%, 56%, and 19%, respectively. Population-weighted distributions were 29%, 77%, and 8%, respectively. Notably, these differences in the distributions of pain conditions had minimal effects on the prevalence of sleep quality, sleep duration, or any of the characteristics described in Table 1. Thus, characteristics of the unadjusted study sample are shown in Table 1. Survey respondents were predominantly female, ages 70 to 79 years, and enrolled in Medicare Supplement plans with high levels of coverage.

**Table 1.** Unadjusted Demographics for Unweighted Study Population by Sleep Quality and Sleep Duration

	Overall	Sleep quality			Sleep duration			
	% or mean	Poor, % or mean	Good, % or mean	P	Short, % or mean	Optimal, % or mean	Long, % or mean	P
**Number**	4201	933	3268	–	1632	2175	394	–
		22.2	77.8	–	38.8	51.8	9.4	–
**Demographic variables**								
Sex								
Female	67.3	72.1	66.0	0.0004	70.3	65.3	65.7	0.004
Male	32.7	27.9	34.1		29.7	34.7	34.3	
Age, years		77.4	78.0	0.02	77.7	77.8	79.2	0.001
65 to 69	10.4	12.9	9.7	0.01	11.4	10.0	8.4	0.007
70 to 74	26.3	27.9	25.8		26.7	26.7	22.1	
75 to 79	25.8	24.3	26.2		25.1	26.9	22.3	
80 to 84	18.7	16.4	19.4		18.0	18.3	24.4	
≥85	18.9	18.5	19.0		18.9	18.2	22.8	
Median Income (from zip code)								
Low	13.8	15.4	13.3	0.30	15.8	12.7	11.9	0.007
Medium	34.8	35.4	34.6		35.3	35.1	31	
High	51.2	49.1	51.9		48.8	52.1	56.6	
Minority (from zip code)								
Low	50.9	50.9	50.9	0.38	49.9	51.8	50.3	0.30
Medium	45.0	44.5	45.2		45.5	44.6	45.4	
High	3.0	3.8	2.8		3.7	2.5	2.8	
Location								
Urban	83.4	83.7	83.3	0.76	83.5	82.7	86.6	0.17
Other	16.6	16.3	16.7		16.5	17.3	13.5	
Region								
Midwest	24.7	23.9	25.0	0.78	23.4	24.9	29.2	0.006
Northeast	19.6	18.5	19.9		20.4	19.6	15.7	
South	26.7	27.4	26.5		28.4	26.3	22.1	
West	28.9	30.0	28.5		27.6	29.1	32.5	
Primary Care Physicians/100,000								
Low	32.5	33.4	32.2	0.9	33.2	32.3	30.5	0.7
Medium	47.7	46.4	47.6		46.3	47.8	49.5	
High	20.0	20.0	20.0		20.4	19.8	19.8	
Plan Type								
High coverage	77.2	76.7	77.4	0.69	76.5	77.2	80.5	0.25
Middle coverage	2.7	2.9	2.6	0.66	2.8	2.5	3.3	0.67
Other	20.1	20.4	20.0	0.81	20.7	20.3	16.2	0.13
**Claims-based variables**								
Dementia	3.6	4.4	3.4	0.14	3.7	2.9	7.4	<0.0001
Sleep Apnea	14.5	14.0	14.6	0.65	14.0	14.5	16.5	0.44
HCC Score								
HCC <0.50	21.3	18.5	22.1	0.005	19.2	23.6	17.3	<0.0001
HCC 0.50 to <1.20	45.6	43.8	46.3		44.9	46.5	43.7	
HCC 1.20 to <2.80	28.3	31.8	27.3		30.8	25.5	33.5	
HCC ≥2.80	4.8	5.8	4.5		5.1	4.5	5.6	
Medications								
Antipsychotics	2.7	4.0	2.3	0.005	3.2	2.1	3.6	0.07
Benzodiazepines	22.1	30.0	19.8	<0.0001	25.5	19.5	22.1	<0.0001
Muscle Relaxants	20.5	25.4	19.1	<0.0001	23.7	18.0	21.1	<0.0001
NSAID	36.3	37.6	35.9	0.32	37.5	35.5	35.5	0.41
Opioid Rx	58.7	67.9	56.0	<0.0001	62.7	55.6	58.6	<0.0001
Sleep Medication Rx	15.5	25.4	12.7	<0.0001	18.3	37.8	13.7	0.0004
**Survey variables**								
Pain condition								
Low Back Pain	56.3	56.9	56.2	0.68	55.8	56.6	57.1	0.83
Osteoarthritis	56.2	57.4	55.8	0.37	56.4	56.2	54.8	0.85
Rheumatoid Arthritis	18.9	20.8	18.4	0.09	20.3	17.7	20.1	0.10
Weight Category								
Normal weight (BMI ≤25)	31.1	28.5	31.8	0.03	29.2	31.9	34.3	0.004
Overweight (BMI 26 to <30)	35.2	34.1	35.5		33.7	35.7	38.1	
Obese (BMI ≥30)	30.7	34.5	29.6		33.8	29.6	23.9	
Depression								
Yes	8.3	17.5	5.7	<0.0001	12.3	4.9	10.4	<0.0001
No	91.0	81.7	93.6		86.6	94.6	88.8	
Perceived stress								
Low Stress	68.2	50.2	73.3	<0.0001	59.6	74.8	67.0	<0.0001
Medium Stress	28.4	41.9	24.5		34.8	23.4	29.7	
High Stress	2.9	7.7	1.5		5.1	1.3	2.5	
Resilience score								
High (score ≥4)	42.3	29.2	46.1	<0.0001	32.4	50.2	39.6	<0.0001
Low (score <4)	57.3	70.7	53.5		67.2	49.5	59.6	
Social Network Index								
Diverse	54.3	45.1	56.9	<0.0001	47.9	59.9	49.8	<0.0001
Medium	26.8	29.6	26.0		28.8	25.0	28.4	
Limited	14.7	21.5	12.8		18.8	10.9	18.8	

Missing response rows were excluded to simplify the table; thus, frequencies may not add to 100%.

BMI, body mass index; HCC, Hierarchical Condition Category; NSAID, nonsteroidal anti-inflammatory drug; Rx, prescription.

### Prevalence of sleep outcomes

More than half of respondents reported at least 1 of the 3 adverse sleep outcomes evaluated in this study. Nearly one quarter of all participants reported poor quality sleep, and more women reported poor quality sleep than did men. Short sleep was the most prevalent sleep issue, with more than one third of respondents reporting that they typically sleep for ≤6 hours per night. Long sleep duration was comparatively less common, with 9% of respondents reporting that they slept ≥10 hours per night. An analysis of the overlap between poor quality and short sleep showed that the majority of poor quality sleepers also were short sleepers, whereas few were long sleepers.

### Perceived stress and depression

Perceived stress and depression were dominant negative risk factors across all 3 poor sleep outcomes. Perceived stress had the largest effect on poor sleep quality (Table 2). Compared to individuals with low levels of stress, those with high stress had nearly 5 times the risk of poor sleep quality, and those with medium stress had >2 times the risk of poor sleep quality. By comparison, depressed individuals had nearly 3 times higher risk of poor sleep quality compared to those without depression.

**Table 2.** Odds Ratios for Reporting Poor Sleep Quality, Estimated Using Multivariable Logistic Regression Models

Variable	Sleep quality			
	Stress model		Depression model	
	Odds ratio	P	Odds ratio	P
Sex (referent: Male)				
Female	1.21	<0.0001	1.23	<0.0001
Age (referent: 65–69 y)				
Age 70–74 y	1.02	0.31	1.04	0.01
Age 75–79 y	1.01	0.45	1.06	0.0004
Age 80–84 y	0.75	<0.0001	0.77	<0.0001
Age ≥85 y	0.88	<0.0001	0.95	0.014
HCC Score (referent: HCC <0.50)				
HCC 0.50 to <1.20	1.11	<0.0001	1.13	<0.0001
HCC 1.20 to <2.80	1.33	<0.0001	1.33	<0.0001
HCC ≥2.80	1.20	<0.0001	1.25	<0.0001
Obesity (referent: BMI <30)				
Obese (BMI ≥30)	1.19	<0.0001	1.17	<0.0001
Medications (referent: not taking)				
Benzodiazepines	1.20	<0.0001	1.22	<0.0001
Muscle Relaxants	1.46	<0.0001	1.41	<0.0001
Opioids	1.30	<0.0001	1.34	<0.0001
Sleep Medications	1.96	<0.0001	1.94	<0.0001
Perceived Stress				
Perceived stress - high	4.86	<0.0001	–	–
Perceived stress - medium	2.21	<0.0001	–	–
Depression (referent: not depressed)				
Depressed (PHQ2 ≥ 3)	–	–	2.50	<0.0001
Resilience (referent: low)				
Resilience - high	0.78	<0.0001	0.70	<0.0001
Social networks (referent: limited)				
Social networks - diverse	0.64	<0.0001	0.64	<0.0001
Social networks - moderate	0.83	<0.0001	0.86	<0.0001

Adjusted for: age, sex, income, minority, region, location, plan type.

BMI, body mass index; HCC, Hierarchical Condition Category; PHQ2, Patient Health Questionnaire-2; y, years.

Individuals with high stress also had 2 times higher risk of reporting short sleep and a 30% increase in the risk of long sleep compared to those reporting low stress (Tables 3 and 4). Likewise, depressed individuals had approximately 2 times higher risk of either short or long sleep duration compared to those without depression.

**Table 3.** Odds Ratios for Reporting Short Sleep Duration, Estimated Using Multivariable Logistic Regression Models

Variable	Short sleep duration			
	Stress model		Depression model	
	Odds ratio	P	Odds ratio	P
Sex (referent: Male)				
Female	1.11	<0.0001	1.11	<0.0001
Age (referent: 65–69 y)				
Age 70–74 y	1.12	<0.0001	1.13	<0.0001
Age 75–79 y	0.99	0.50	1.01	0.43
Age 80–84 y	0.84	<0.0001	0.85	<0.0001
Age ≥85 y	0.87	<0.0001	0.90	<0.0001
HCC Score (referent: HCC <0.50)				
HCC 0.50 to <1.20	1.26	<0.0001	1.26	<0.0001
HCC 1.20 to <2.80	1.41	<0.0001	1.41	<0.0001
HCC ≥2.80	1.25	<0.0001	1.26	<0.0001
Obesity (referent: BMI <30)				
Obese (BMI ≥30)	1.17	<0.0001	1.16	<0.0001
Medications (referent: not taking)				
Benzodiazepines	1.22	<0.0001	1.22	<0.0001
Muscle Relaxants	1.36	<0.0001	1.34	<0.0001
Opioids	1.15	<0.0001	1.17	<0.0001
Perceived Stress (referent: low stress)				
Perceived stress - high	2.19	<0.0001	–	–
Perceived stress - medium	1.53	<0.0001	–	–
Depression (referent: no depression)				
Depression - PHQ2 ≥ 3	–	–	1.92	<0.0001
Resilience (referent: low)				
Resilience - high	0.62	<0.0001	0.60	<0.0001
Social networks (referent: limited)				
Social networks - diverse	0.68	<0.0001	0.70	<0.0001
Social networks - moderate	0.73	<0.0001	0.75	<0.0001

Adjusted for: age, sex, minority, region, location, plan type.

BMI, body mass index; HCC, Hierarchical Condition Category; PHQ2, Patient Health Questionnaire-2; y, years.

**Table 4.** Odds Ratios for Reporting Long Sleep Duration, Estimated Using Multivariate Logistic Regression Modeling

Variable	Long sleep duration			
	Stress model		Depression model	
	Odds ratio	P	Odds ratio	P
Sex (referent: Male)				
Female	0.89	<0.0001	0.90	<0.0001
Age (referent: 65–69 y)				
Age 70–74 y	1.02	0.52	1.04	0.11
Age 75–79 y	0.88	<0.0001	0.90	0.0001
Age 80–84 y	1.44	<0.0001	1.45	<0.0001
Age ≥85 y	1.22	<0.0001	1.23	<0.0001
HCC Score (referent: HCC <0.50)				
HCC 0.50 to <1.20	1.05	0.006	1.05	0.006
HCC 1.20 to <2.80	1.32	<0.0001	1.32	<0.0001
HCC ≥2.80	1.31	<0.0001	1.25	<0.0001
Obesity (referent: BMI <30)				
Obese (BMI ≥30)	0.65	<0.0001	0.63	<0.0001
Medications (referent: not taking)				
Antipsychotics	2.34	<0.0001	2.15	<0.0001
Benzodiazepines	1.30	<0.0001	1.24	<0.0001
Opioids	1.01	0.41	0.99	0.48
Sleep Medications	0.85	<0.0001	0.76	<0.0001
Perceived Stress (referent: low stress)				
Perceived stress - high	1.31	<0.0001	–	–
Perceived stress - medium	1.28	<0.0001	–	–
Depression (referent: no depression)				
Depression - PHQ2 ≥ 3	–	–	2.17	<0.0001
Resilience (referent: low)				
Resilience - high	0.73	<0.0001	0.76	<0.0001
Social networks (referent: limited)				
Social networks - diverse	0.68	<0.0001	0.74	<0.0001
Social networks - moderate	0.57	<0.0001	0.61	<0.0001

Adjusted for: age, sex, minority, region, location, plan type.

BMI, body mass index; HCC, Hierarchical Condition Category; PHQ2, Patient Health Questionnaire-2; y, years.

### Prescription medications

As an indicator of pain, opioids were the most commonly used prescription medications. Opioid use was associated with a 30% increased risk of poor quality sleep, and an approximate 15% increased risk for short sleep duration. Opioids were not significantly associated with long sleep duration.

Sleep-inducing medications also were common, with 22% using benzodiazepines and 16% using sleep medications. Sleep medication users had nearly 2 times higher risk of poor sleep quality and a 20% reduced risk of long sleep. Benzodiazepine use was associated with a 20% increased risk of both poor sleep quality and short sleep and a 30% increased risk of long sleep. Use of antipsychotic medications was associated with >2 times increased risk of long sleep duration.

### Resilience and social networks

Resilience and social networks were associated with significantly reduced odds for all 3 sleep outcomes. Compared to low resilience, high resilience was associated with a 40% reduction in the odds of poor sleep quality. Compared to those with limited social networks, having a moderate or diverse social network was associated with 20% to 40% reduced odds for all 3 sleep outcomes.

## Discussion

In this population of AARP Medicare Supplement insureds with chronic pain, short sleep duration was the most common sleep complaint, followed by poor sleep quality, and long sleep duration. Perceived stress, depression, and prescription medications were independently associated with all 3 poor sleep outcomes. Positive psychosocial factors, including higher resilience and more diverse social networks, were strongly associated with better sleep outcomes among older adults with chronic pain.

More than half of survey respondents reported problems related to sleep quality or duration. Though prevalence rates vary, this is generally consistent with findings from large epidemiological studies of community-dwelling older adults. For example, 57% of participants reported chronic sleep complaints in an observational study of more than 9000 community-dwelling older adults in the United States.^33^ Notably, the prevalence of any sleep complaint declined to 39% after excluding individuals with depression or physical limitations.^33^ Thus, the high prevalence of sleep problems observed among survey respondents is unsurprising considering all study participants had at least 1 chronic pain condition.

Poor sleep quality was reported by 22% of survey respondents, which aligns closely with findings from the 2003 NSF Sleep in America survey of 1500 community-dwelling older adults ages 55 to 84. The study reported declining sleep quality with an increasing number of coexisting medical conditions. Specifically, poor sleep quality was found among 41%, 22%, and 10% of participants with ≥4, 1 to 3, or no coexisting medical conditions, respectively.^2^

Among survey respondents, 38% and 9% reported short and long sleep durations, respectively. In contrast, 22% and 7% of older adults who participated in the National Health and Nutrition Examination Survey (NHANES) reported short or long sleep durations, respectively.^34^ Although the definitions for short and long sleep duration were the same in both studies and all participants in the NHANES study were ages ≥75 years, nearly 75% described their health status as “good” to “excellent.” When NHANES participants with mild to severe depressive symptoms were examined independently, however, 35% reported short sleep duration and more than 20% reported long sleep duration. Future studies could further investigate the potential effects of pain on the relationship between depression and long sleep duration.

Multivariate analyses revealed strong relationships between psychological and psychosocial factors and sleep in older adults. Consistent with previous studies, poor psychological health, specifically depression or medium to high levels of perceived stress, were strongly associated with poor sleep quality and duration. Notably, high levels of perceived stress had nearly 5 times greater risk of poor sleep quality and more than twice the risk of short sleep duration. Even at medium levels, perceived stress was associated with twice the risk of poor sleep quality and a 50% increased risk of short sleep. Depression also was strongly associated with 2.5 times greater risk of poor sleep quality and approximately twice the risk of either short or long sleep duration. These results corroborate prior reports that anxiety is more closely associated with sleep than depression and highlight the importance of stress as an insidious health risk across the life span.^15^

Higher resilience and moderate to diverse social networks were associated with reduced risk of poor sleep quality and short or long sleep duration. Prior sleep research among older adults has focused mainly on the negative physical and psychological aspects associated with poor quality sleep and long or short sleep duration. Because of reciprocal associations between sleep, pain, depression, and anxiety, sleep problems can worsen other medical conditions.^1,3,18^ Thus, poor sleep worsens pain, which can contribute to increased depression and stress, thereby worsening sleep, and so on. Because resilience and social networks work in opposition to this feedback loop, this study suggests that strategies to support positive psychosocial characteristics also might improve sleep. Likewise, improvements in sleep health also may reinforce positive psychosocial well-being among older adults.

Long sleep duration was most strongly associated with antipsychotic medication use. Despite a “black box” warning and an increased risk of all-cause mortality, antipsychotics are often prescribed off label to address behavioral, psychological, or sleep symptoms, particularly in dementia patients.^35^ This association likely reflects the known sedating effects of antipsychotics and the clinical complexity of elderly patients who are prescribed antipsychotics off label.

More than half of this population used opioids, and nearly one quarter used benzodiazepines or muscle relaxants. This is consistent with the use of opioids in older adults with either back pain or combinations of back pain and osteoarthritis.^36,37^ Concurrent use of opioids with benzodiazepines, muscle relaxants, and sleep medications is contraindicated for management of pain and sleep problems in older adults.^19,38^ Thus, clinicians should take care to avoid prescribing opioids and sedatives to older adults who have both pain and sleep problems. Results from multivariate analyses showed that the risk of poor quality sleep and both short and long sleep duration was associated with the use of these medications.^9,19^ However, as this is a cross-sectional study, the authors cannot determine whether this increased risk is attributable to the use of these medications per se versus an indication that pain and sleep problems are not fully managed by the use of these medications. In any case, the use of opioids does not necessarily imply that symptoms are adequately managed. Ultimately, more non-pharmacological alternatives to improve sleep and manage pain are needed but currently not readily accessible or practical because of time and resource limitations.

Cognitive behavioral therapy (CBT) and mindfulness-based interventions are leading non-pharmacological intervention approaches that include strategies to reduce the harmful effects of stress and depression while also promoting positive psychosocial attributes.^36^ CBT and sleep hygiene interventions are considered first-line therapies for sleep problems in older adults.^19,38^ Despite demonstrated efficacy, mental health services typically are underutilized by older adults, and physicians rarely refer patients with sleep problems for CBT.^38,39^ When referred, many older adults report feeling uncomfortable having discussions about personal issues with a health professional.^39^ In addition, CBT requires time and commitment from both the patient and provider for the benefits to be realized.^19^ Because mindfulness-based interventions do not require a licensed therapist and have demonstrated efficacy in improving sleep, these may be a more feasible treatment option.

### Limitations and strengths

Limitations of this study include an inability to evaluate causation because of the cross-sectional design. In addition, this study relied on self-reported sleep quality and duration, which may be less reliable than objective sleep measurements such as actigraphy or polysomnography. Finally, findings from this population of AARP Medicare Supplement insureds may not be generalizable to other Medicare Supplement insureds.

Strengths of this research include the use of a large sample of older adults alongside a comprehensive combination of variables, in a large sample of older adults with chronic pain. This study draws on prior research by social and behavioral health scientists who have long indicated that sleep is “embedded within the social world.”^40^ This study contributes to the understanding of sleep in older adults by broadening the scope of research through a comprehensive evaluation of factors from multiple domains, including physical, psychological, and psychosocial contributors.

## Conclusions

In summary, more than half of the older adults with chronic pain who were evaluated in this study reported at least 1 sleep problem. Out of the comprehensive list of variables, sleep quality and duration were associated most strongly with psychological and psychosocial factors. The strongest predictors of poor sleep quality and short sleep duration included medium and high levels of perceived stress and depression. Use of opioids, benzodiazepines, and muscle relaxants was common in this population and associated with poor sleep outcomes. Conversely, positive psychosocial characteristics, including high resilience and diverse social networks, had strong protective effects. These findings indicate that greater resilience and social networks may provide opportunities for new ways to improve sleep and well-being in older adults with chronic pain.
